# Synergistic Zn-Cd Bimetallic Engineering in ZIFs for High-Chloride 2e^−^ ORR to H_2_O_2_ in Simulated Neutral Seawater

**DOI:** 10.3390/ma18081786

**Published:** 2025-04-14

**Authors:** Xu Wang, Nan Wang, Kunpeng Liu, Meinan Yang, Ruiyong Zhang, Sikandar Khan, Jinhui Pang, Jizhou Duan, Baorong Hou, Wolfgang Sand

**Affiliations:** 1 College of Biological Engineering, Qingdao University of Science and Technology, Qingdao 266042, China18090654697@163.com (M.Y.); 2 State Key Laboratory of Advanced Marine Materials, Institute of Oceanology, Chinese Academy of Sciences, Qingdao 266071, China; wangnan123@qdio.ac.cn (N.W.);; 3 Department of Biotechnology, Shaheed Benazir Bhutto University, Sheringal 18000, Pakistan

**Keywords:** oxygen reduction reaction, neutral condition, electrocatalysis, H_2_O_2_, seawater

## Abstract

Marine biofouling causes significant economic losses, and conventional antifouling methods are often associated with environmental pollution. Hydrogen peroxide (H_2_O_2_), as a clean energy source, has gained increasing attention in recent years. Meanwhile, electrocatalytic 2e^−^ oxygen reduction reaction (ORR) for H_2_O_2_ production has received growing interest. However, the majority of current studies are conducted on acidic or alkaline electrolytes, and research on 2e^−^ ORR in neutral NaCl solutions remains rare. Here, a bimetallic Zn-Cd zeolitic imidazolate framework (ZnCd-ZIF) is rationally designed to achieve chloride-resistant 2e^−^ ORR catalysis under simulated seawater conditions (pH 7.5, 3.5% Cl^−^). Experimental results demonstrate that the ZnCd-ZIF catalyst exhibits an exceptional H_2_O_2_ selectivity of 70% at 0.3 V_RHE_, surpassing monometallic Zn-ZIF (60%) and Cd-ZIF (50%). Notably, H_2_O_2_ production reaches 120 mmol g^−1^ in a Cl^−^-containing neutral electrolyte, exhibiting strong resistance to structural corrosion and Cl^−^ poisoning. This work not only pioneers an effective strategy for designing ORR catalysts adapted to marine environments but also advances the practical implementation of seawater-based electrochemical H_2_O_2_ synthesis.

## 1. Introduction

Microbial fouling in marine environments has long hindered the sustainable development of economic and offshore engineering sectors [1,2]. Traditional antifouling technologies rely mainly on chemical biocides such as organotin and cuprous oxide [2]. However, the ecotoxicity caused by their toxic residues has been strictly restricted by international conventions, such as the AFS Convention [3]. Hydrogen peroxide (H_2_O_2_), known as a “green biocide” due to its decomposition into water and oxygen alone, exhibits unique advantages in in situ marine disinfection [3]. However, the current H_2_O_2_ supply model relies heavily on centralized chemical plant production via the anthraquinone process, followed by long-distance transportation to the point of use. This fundamentally contradicts the decentralized, energy-efficient requirements of marine scenarios [4]. An electrocatalytic oxygen reduction reaction (ORR) provides a new approach to address this contradiction through in situ H_2_O_2_ synthesis: using seawater as a natural electrolyte and renewable energy to drive two-electron oxygen reduction, an “on-demand production” antifouling system can be established [5]. In the context of global sustainable development, the electrochemical two-electron oxygen reduction reaction (2e^−^ ORR) has emerged as a sustainable alternative for decentralized H_2_O_2_ production. However, challenges in selectivity persist, primarily due to competition with the dominant 4e^−^ ORR pathway used in fuel cells [6].

Metal–organic frameworks (MOFs) offer unique advantages for electrochemical ORR due to their tunable porosity, high surface area, and atomic-level designability. These features enable the precise regulation of active sites, mass transport properties, and electronic structures [7], which are critically aligned with the requirements of 2e^−^ ORR. However, despite the advantages of MOFs, under neutral pH conditions, MOF-based catalysts face intrinsic challenges, including weak proton availability for intermediate stabilization, the competitive adsorption of non-reactive ions (e.g., Cl^−^ in seawater), and limited electrochemical stability under prolonged polarization [8,9].

Neutral pH systems have gained attention as a promising frontier due to their applications in seawater electrolysis and biomedical systems [10,11]. However, research on 2e^−^ ORR catalysts under neutral conditions remains limited, particularly in complex systems like natural seawater and simulated seawater [12]. Concurrently, high concentrations of chloride ions (Cl^−^) can induce corrosion on the catalyst surface, further limiting its catalytic performance [13,14].

Recent advancements in MOFs—such as ZIF-8 derivatives and Fe-PCN-224—have shown progress through strategies like metal doping and ligand functionalization [15,16]. However, critical challenges remain: (1) achieving both a high H_2_O_2_ yield and long-term stability [17], (2) the insufficient exploration of eco-friendly MOF synthesis utilizing waste-to-resource conversion, and (3) a lack of systematic studies on structure–activity relationships under neutral conditions [18]. It is of great significance to realize a high two-electron ORR selectivity under natural conditions by modifying the catalyst and to offer promising applications in offshore H_2_O_2_ generation via seawater electrolysis and the production of biocompatible disinfectants. Nevertheless, research on 2e^−^ ORR is primarily focused on acidic and alkaline conditions, with limited studies exploring 2e^−^ ORR using high-chloride simulated seawater as the electrolyte [19]. Recent research indicates that Zn can effectively facilitate the 2e^−^ ORR process under neutral conditions, and Cd doping in Zn further enhances catalyst dynamics [20,21].

In this study, ZnCd-ZIF with different molar ratios was synthesized as an ideal catalyst for electrocatalytic 2e^−^ ORR in simulated seawater. The structural and elemental distributions of ZnCd-ZIF were characterized using SEM, XRD, and XPS. Electrochemical measurements were conducted to determine its electrocatalytic performance. H_2_O_2_ production tests and stability evaluations were performed in simulated seawater. These results demonstrate the practical feasibility of the catalyst for real-world applications and pave the way for subsequent studies in natural seawater.

## 2. Materials and Methods

### 2.1. Materials

The chemicals used in this study include zinc nitrate hexahydrate (Shanghai Aladdin Bio-Chem Technology Co., Ltd., Shanghai, China), cadmium nitrate tetrahydrate (Sinopharm Chemical Reagent Co., Ltd., Beijing, China), 2-methylimidazole, cetyltrimethylammonium bromide, ethanol, and methanol (Shanghai Chemical Reagent Co., Ltd., Shanghai, China). All water used in the experiment was deionized. Experimental reagents and chemicals were used without further purification.

### 2.2. Preparation of ZnCd-ZIF Catalysts with Different Doping Proportions

Zn-ZIF: Zn-ZIF was synthesized by dissolving 10.08 g of 2-methylimidazole in 170 mL of deionized water, followed by the addition of 4.5 mL of a 0.01 M aqueous solution of cetyltrimethylammonium bromide (CTAB) with stirring for 5 min. Subsequently, 30 mL of a Zn(NO_3_)_2_·6H_2_O solution was introduced into the mixture, which was stirred at room temperature for 4 h. The resulting product was collected by centrifugation, washed three times with methanol, and dried at 60 °C for 5 h.

Cd-ZIF: Cd-ZIF was synthesized following the same procedure as described above, with Zn(NO_3_)_2_·6H_2_O replaced by Cd(NO_3_)_2_·4H_2_O.

The synthesis of ZnCd-ZIF materials with varying ratios solely involves adjusting the molar ratio of raw materials, while all other parameters are kept consistent. All samples were calcined at 800 °C in an Ar atmosphere, with a heating ramp of 1 h, followed by calcination for an additional 1 h. And the heating ramp rate was 5 °C per minute.

### 2.3. Characterization Equipment

A scanning electron microscope (SEM, HITCH, Tokyo, Japan) and a transmission electron microscope (TEM, JEOL-F200, Tokyo, Japan) were used to analyze the crystal structure and micromorphology of the samples. The chemical state of the samples was examined using X-ray photoelectron spectroscopy (XPS, ESCALAB Xi+, Thermo Fisher Scientific, Waltham, MA, USA). The UV–vis absorption spectra were recorded using a UV–vis spectrophotometer (HITCH 3900, HITCH, Tokyo, Japan).

### 2.4. Electrochemical Measurements

Electrochemical tests were performed using a CHI 760E electrochemical workstation (Shanghai Chenhua Instrument Co., Shanghai, China) equipped with a rotating ring-disk electrode (RRDE, Pine, Durham, NC, USA). The three-electrode system consisted of a saturated calomel electrode (SCE) as the reference electrode, a graphite rod as the counter electrode, and a rotating disk electrode with a disk diameter of 0.2415 cm^2^. Catalyst ink was prepared by dispersing the material in isopropyl alcohol containing 1 μL of 5% Nafion, achieving a mass concentration of 1 mg mL^−1^. Subsequently, 6 μL of each catalyst ink was drop-cast onto pre-cleaned glassy carbon disk electrodes and air-dried at room temperature. The catalyst-modified working electrodes underwent cyclic voltammetry (CV) measurements at a scan rate of 100 mV s^−1^ in argon-saturated 3.5% NaCl.

The oxygen reduction reaction (ORR) catalytic activity was evaluated using a rotating ring-disk electrode system in an oxygen-saturated electrolyte with solution resistance compensation at 25 °C, a rotation speed of 1600 rpm, and a scan rate of 10 mV s^−1^. ORR catalytic selectivity was assessed by polarizing the platinum ring electrode to 1.3 V_RHE_ to oxidize the hydrogen peroxide (H_2_O_2_) generated at the disk electrode. The electron transfer number and H_2_O_2_ selectivity were determined from the catalyst’s rotating ring-disk electrode voltammograms using the following equations (Equations (1) and (2)).

(1) $$n=\frac{4i_{d}}{i_{d}+i_{r}/N}$$

(2) $$H_{2}O_{2}=200\times \frac{i_{r}/N}{\left(i_{d}+i_{r}/N\right)}$$

i_d_, disk current. i_r_, ring current. N = 0.37, the current collection efficiency of the Pt-ring.

### 2.5. Detection of H_2_O_2_ Production

Hydrogen peroxide (H_2_O_2_) production was measured in a two-compartment electrolytic cell separated by a Nafion 117 membrane. Catalysts were deposited onto carbon paper substrates, and 30 mL of 3.5% NaCl was added to both the anode and cathode compartments.

The H_2_O_2_ concentration was quantified using a cerium sulfate (Ce(SO₄)_2_) titration method, based on a redox reaction in which yellow Ce^4+^ is reduced to colorless Ce^3+^ by H_2_O_2_ (Equation (3)) [22,23]. UV–vis spectrophotometry at 318 nm was employed to track the absorbance changes of the Ce^4+^ before and after the reaction. The H_2_O_2_ yield was calculated using Equation (4), where M_Ce_^4+^ represents the molar amount of consumed Ce^4+^.

(3) $$2{Ce}^{4+}+H_{2}O_{2}\to 2{Ce}^{3+}+2H^{+}+O_{2}$$

(4) $$M=\frac{1}{2}\times M_{{Ce}^{4+}}$$

## 3. Results and Discussion

### Characterizations of Catalysts

**Figure 1:**
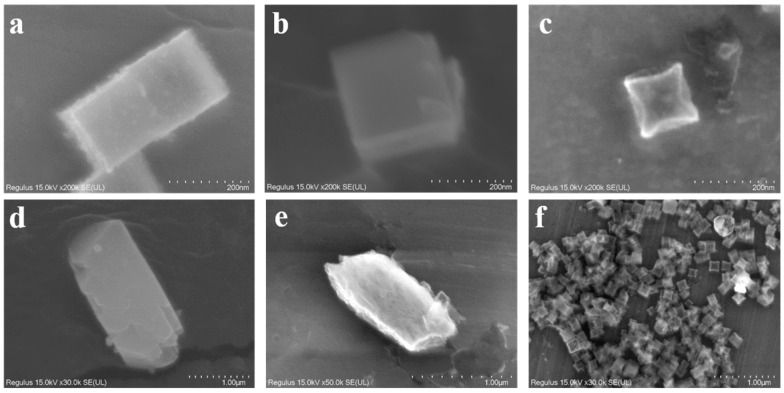
SEM images of (**a**) Zn-ZIF, (**b**) ZnCd-ZIF (4:1), (**c**) ZnCd-ZIF (1:1), (**d**) ZnCd-ZIF (1:4), and (**e**) Cd-ZIF, (**f**) ZnCd-ZIF (4:1). All SEM images are at the scale of 1 µm.

As outlined in the synthetic methodology, controlled compositional tuning through elemental ratio variation was employed to develop distinct ZIF-type catalyst variants with a systematic architectural evolution. To investigate the morphological transformations induced by varying Cd doping ratios, SEM characterization was performed (Figure 1). Figure 1a presents the Zn-ZIF (ZIF-8) sample without Cd doping, exhibiting a well-defined cubic architecture characteristic of standard ZIF-8, confirming successful precursor synthesis. In contrast, the ZnCd-ZIF (4:1) sample (Figure 1b) retains fundamental cubic features but shows signs of partial structural collapse. This collapsing tendency intensifies with increasing Cd content, as evident in Figure 1c. At a Zn:Cd ratio of 1:4 (Figure 1d), the morphology transitions into prismatic structures, with particle sizes expanding to approximately 1.5 μm. The fully Cd-substituted ZIF sample (Figure 1e) exhibits a complete loss of angular features, with a more fragmented surface. A higher Cd content induces a morphological transition of the material from Zn-ZIF to Cd-ZIF. Additionally, as shown in Figure 1f, the synthesized catalyst exhibits a uniform distribution and consistent size, with an average diameter of approximately 150 nm. This effectively demonstrates the successful synthesis of the material.

**Figure 2:**
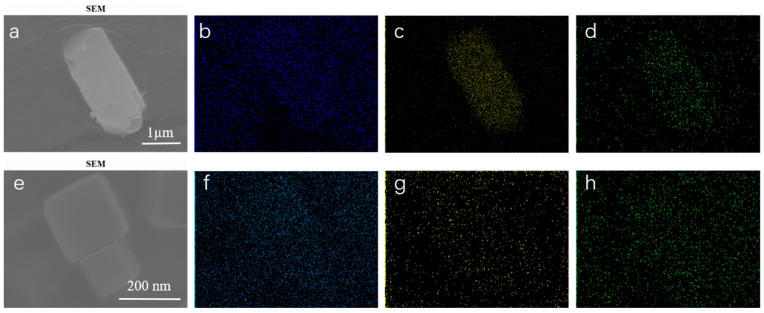
(**a**) SEM image of ZnCd-ZIF (1:4), (**b**–**d**) mapping of oxygen, cadmium, and zinc elements, respectively. (**e**) SEM image of ZnCd-ZIF (4:1) and (**f**–**h**) mapping of oxygen, cadmium, and zinc elements, respectively.

To further confirm the elemental composition of the ZIF catalysts, Energy-Dispersive X-ray Spectroscopy (EDS) and elemental mapping analyses were performed on ZnCd-ZIF (1:4) and ZnCd-ZIF (4:1) samples (Figure 2). The results indicate that oxygen, cadmium, and zinc are uniformly distributed within the composite material, verifying the successful synthesis of the catalysts ZnCd-ZIF (1:4) and ZnCd-ZIF (4:1). As expected, variations in doping ratios influence the relative intensity of elemental mapping signals; however, all elements remain homogeneously dispersed throughout the structure.

**Figure 3:**
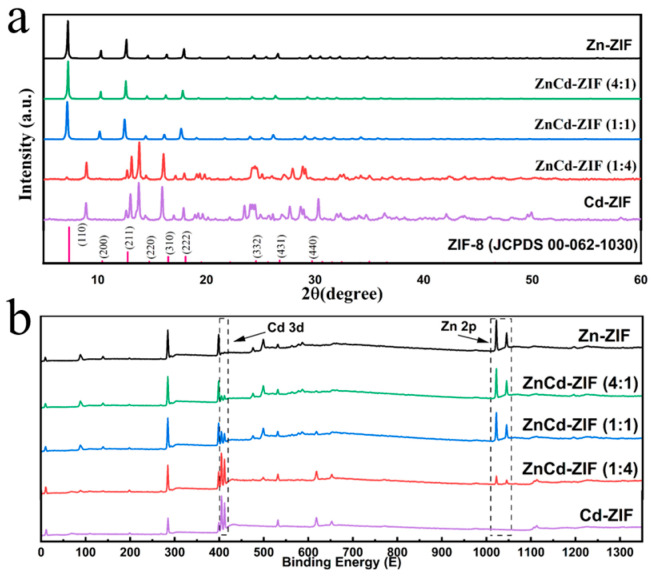
(**a**) XRD spectra of catalysts with varying Zn/Cd ratios. (**b**) XPS survey scan highlighting Cd and Zn peak positions, along with XPS survey scans of catalysts with different Zn/Cd ratios.

Figure 3 presents the X-ray diffraction analysis (XRD) and XPS characterization profiles of the synthesized catalysts. XRD analysis was systematically conducted to elucidate the crystalline structures and atomic arrangements of catalysts with varying Cd doping ratios. As shown in Figure 3a, the diffraction patterns of Zn-ZIF align well with the standard JCPDS cards of ZIF-8 (00-062-1030), where all crystal planes (e.g., (110), (211)) showed good correspondence, confirming the successful synthesis of highly crystalline Zn-ZIF frameworks. Notably, upon Cd’s incorporation into the Zn-ZIF matrix to form bimetallic ZnCd-ZIF catalysts, the characteristic diffraction peaks at 10.28° and 17.98° exhibit discernible low-angle shifts to 10.19° and 17.67°, respectively. This shift is attributed to the larger ionic radius of Cd^2+^ compared to Zn^2+^, inducing lattice expansion. The XRD data collectively confirm the successful formation of bimetallic ZIFs. As the Cd content increases, a progressive deviation from the simulated Zn-ZIF diffraction pattern becomes evident, with ZnCd-ZIF (1:4) and Cd-ZIF displaying analogous diffraction patterns.

A complementary XPS analysis (Figure 3b) was conducted to investigate chemical composition variations induced by Cd doping. The survey spectra revealed composition-dependent electronic state changes; Zn2p orbital signals (1020–1050 eV) progressively weakened and became undetectable as the Zn content decreased, while Cd3d orbital signals (402–414 eV) intensified correspondingly. This inverse signal correlation conclusively validates the successful synthesis of bimetallic ZIF catalysts with precisely controlled elemental ratios.

**Figure 4:**
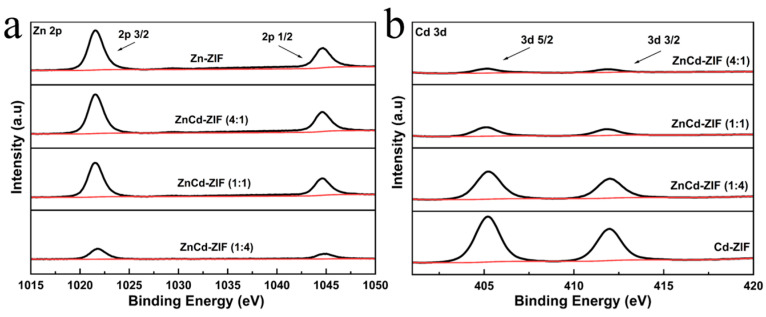
(**a**) Comparison of XPS spectra for the Zn2p orbitals. (**b**) Comparison of XPS spectra for the Cd3d orbitals.

In order to more clearly demonstrate the XPS results, a comparison of the peak shapes of the orbits of Zn and Cd is shown in Figure 4. Figure 4a shows the core-level spectra of Zn 2p in the X-ray photoelectron spectroscopy (XPS) of catalysts with different ratios. This spectrum exhibited characteristic peaks at 1022 eV and 1044.5 eV, corresponding to Zn 2p3/2 and Zn 2p1/2, respectively. It indicated the existence of a divalent oxidation state in the sample [24,25]. Different radios of ZnCd-ZIF showed a slight chemical shift in the binding energy compared with ZIF-8 because of the presence of Cd. The XPS spectrum of Cd 3d shows that the two strong peaks at about 405.5 eV and 412 eV are related to Cd 3d5/2 and Cd 3d3/2 in Figure 4b, respectively, demonstrating the Cd^2+^ of the catalysts [26,27]. Concurrently, the enhancement of cadmium-associated spectral signals correlates directly with elevated Cd concentrations in the catalytic material.

**Figure 5:**
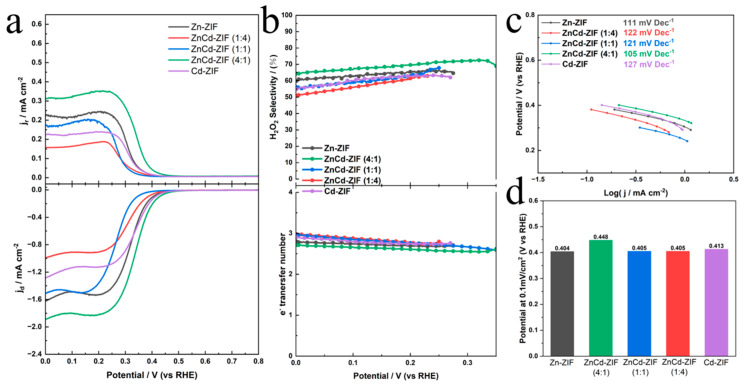
(**a**) ORR performance of ZnCd-ZIF catalysts with different doping ratios in 3.5% NaCl. (**b**) Calculated H_2_O_2_ selectivity at various applied potentials and electron-transfer numbers during a potential sweep. (**c**) Tafel slopes plotted from the H_2_O_2_ ring current, and (**d**) onset potentials derived from polarization curves.

The electrocatalytic optimization of the two-electron oxygen reduction reaction (2e^−^ ORR) fundamentally relies on enhancing active site performance. Comprehensive electrochemical evaluations employing a three-electrode configuration were conducted in simulated seawater (3.5% NaCl) to assess ORR catalytic efficiencies across differently doped catalysts, with performance metrics detailed in Figure 5. Prior to testing, all catalysts underwent systematic activation via cyclic voltammetry (CV) in an oxygen-saturated electrolyte. Subsequent steady-state linear sweep voltammetry (LSV) measurements (Figure 5a) demonstrated a significant enhancement in 2e^−^ ORR activity for ZnCd-ZIF (4:1) compared to pristine Zn-ZIF. A critical analysis of disk and ring current differentials enabled the precise calculation of electron transfer numbers (n) and H_2_O_2_ selectivity gradients. The earlier onset of disk current in ZnCd-ZIF (4:1) (Figure 5a) signifies accelerated activation kinetics; however, rigorous validation with concurrent ring current profiles was imperative to verify that the catalyst can reduce the electron transfer number and enhance H_2_O_2_ selectivity.

Quantitative selectivity parameters in Figure 5b demonstrate that ZnCd-ZIF (4:1) exhibited a superior and progressively escalating H_2_O_2_ selectivity (~65–70%) with a lower electron transfer number. In contrast, other doping ratios exhibited a reduced selectivity (50–60%) and elevated electron transfer numbers. According to the experimental results, both 2e^−^ and 4e^−^ pathways proceed concurrently during the electrocatalytic oxygen reduction process. ZnCd-ZIF (4:1)’s decrease in the electron transfer number indicates an enhanced tendency of the catalyst towards the 2e^−^ reaction pathway, thereby improving its selectivity. Tafel analysis (Figure 5c) further supports these findings, as ZnCd-ZIF (4:1) achieved the lowest kinetic barrier at 105 mV dec^−1^, compared to 111–127 mV dec^−1^ for other catalysts. The onset potential metric (defined at a 0.1 mA cm^−2^ current density, Figure 5d) was determined to be 0.448 V_RHE_ for ZnCd-ZIF (4:1), indicating a favorable electrolytic response. Collectively, these results establish the Zn:Cd ratio of 4:1 as the optimal composition for efficient 2e^−^ ORR catalysis in chloride-containing media.

The integrated electrochemical characterization results demonstrate that catalyst selectivity and reaction kinetics can be comprehensively analyzed through multidimensional test systems. The peroxide reduction reaction (PRR) activity is a key factor in determining the net H_2_O_2_ generation during electrocatalysis. In our study, we investigated the PRR by performing cyclic voltammetry (CV) in an Ar-saturated 3.5% NaCl solution with and without 10 mM H_2_O_2_. After the introduction of H_2_O_2_, the CV curves showed no significant increase in redox current, indicating that the catalyst did not promote the further decomposition of H_2_O_2_.

**Figure 6:**
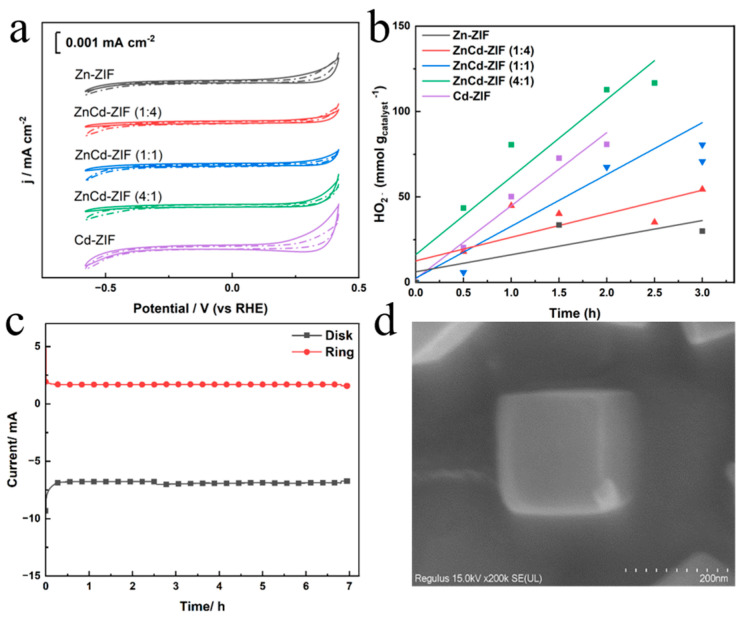
(**a**) CV curve in Ar-saturated 3.5% NaCl containing 10 mM H_2_O_2_ (solid lines) and without 10 mM H_2_O_2_ (dashed lines), (**b**) H_2_O_2_ production amount normalized to catalyst loading amount over reaction time, (**c**) stability test, (**d**) SEM image of ZnCd-ZIF (4:1) after stability test.

The quantification of H_2_O_2_ production rates, an essential component of electrochemical evaluation, was further investigated using an H-cell configuration (Figure 6b). Under potentiostatic operation for 3 h, ZnCd-ZIF (4:1) achieved a cumulative H_2_O_2_ yield of approximately 120 mmol g^−1^, significantly surpassing the performance of other catalysts. These quantitative results unequivocally validate the superior enhancement of ZnCd-ZIF (4:1) in promoting the 2e^−^ ORR pathway. Long-term cycling stability was evaluated using chronoamperometric tests at 0.3 V_RHE_ in an O_2_-saturated 3.5% NaCl solution with a 1600 rpm rotation (Figure 6c). Over a 7 h continuous operation, both disk and ring current responses for ZnCd-ZIF (4:1) gradually stabilized, demonstrating exceptional electrochemical endurance under operational conditions. In Figure 6d, we conduct a post-cycling morphological characterization, which shows that the catalyst’s basic structure remained intact, confirming its structural stability. Based on our experimental characterization, the proposed ORR mechanism involves the dissociation of the O-O bond after oxygen adsorption on ZnCd-ZIF due to its strong oxygen adsorption capability [28]. This promotes O_2_ adsorption and reaction with water to form the *OOH intermediate, which then desorbs to generate H_2_O_2_ through the 2e^−^ ORR pathway [29]. This indicates that ZnCd-ZIF enhances both the selectivity and activity of the 2e^−^ reduction to H_2_O_2_ by optimizing *OOH binding energy.

Finally, we compare the ORR performance of ZnCd-ZIF with the reported catalysts in Table 1. In neutral solutions, this ZnCd-ZIF catalyst exhibits balanced and superior performance in terms of onset potential and H_2_O_2_ yield, while also demonstrating excellent stability.

## 4. Conclusions

ZIF-8 catalysts with varying doping ratios (1:4, 1:1, 4:1) were synthesized in this study. Among all of them, the ZnCd-ZIF (4:1) catalyst exhibited a superior catalytic activity in neutral simulated seawater. This catalyst demonstrated a higher onset potential (Eonset = 0.448 V_RHE_) and hydrogen peroxide (H_2_O_2_) selectivity (~70%) compared to those with other doping ratios. Furthermore, ZnCd-ZIF (4:1) exhibited robust stability, achieving a cumulative H_2_O_2_ yield of 120 mmol g^−1^. The incorporation of cadmium in ZnCd-ZIF (4:1) not only enhanced its electrocatalytic ORR performance but also facilitated the environmentally responsible recycling of this toxic element, presenting a novel approach for green recycling processes in line with sustainability goals.

## Figures and Tables

**Table 1:** A comparative summary of H_2_O_2_ production performance with other catalysts.

Electrocatalysts	Electrolytes	H_2_O_2_ Yield (%)	Onset Potential	Stability (h)	Reference
Nb_2_O_5_-rGO	0.1 M K_2_SO_4_	85.3%	0.300 V_RHE_	5	[30]
NCMK3IL50-800T	0.1 M K_2_SO_4_	55–85%	0.450 V_RHE_	-	[31]
GO_X_/MnCO_3_	3.5% NaCl	50%	0.635 V_RHE_	-	[29]
ZnO/rGO	0.5 M NaCl	75–78%	0.335 V_RHE_	6	[4]
rGO	0.5 M NaCl	56–60%	0.409 V_RHE_	6	[4]
ZnCd-ZIF	3.5% NaCl	65–70%	0.448 V_RHE_	7	this work

## Data Availability

The original contributions presented in the study are included in the article; further inquiries can be directed to the corresponding authors.
